# Discrepancies between primary and secondary interpretations of pediatric nuclear medicine imaging examinations

**DOI:** 10.1007/s00247-025-06441-w

**Published:** 2025-12-04

**Authors:** Peter Hoeksema, Shireen Hayatghaibi, Susan E. Sharp, Yinan Li, Christopher G. Anton, Robin E. Norris, Andrew T. Trout

**Affiliations:** 1 Department of Radiology, Cincinnati Children’s Hospital Medical Center, 3333 Burnet Avenue, MLC 5031, Cincinnati, 45229 OH USA; 2 Department of Radiology, Corewell Health William Beaumont University Hospital, Royal Oak, MI USA; 3 Department of Radiology, University of Cincinnati, Cincinnati, USA; 4 Division of Oncology, Cancer and Blood Diseases Institute, Cincinnati Children’s Hospital Medical Center, Cincinnati, USA; 5 Department of Pediatrics, University of Cincinnati, Cincinnati, USA

**Keywords:** Discrepancies, Imaging, Pediatric nuclear medicine

## Abstract

**Background:**

Requests for secondary interpretation of imaging examinations adds clinical work and generates additional charges.

**Objective:**

To understand the impact of secondary interpretations of pediatric nuclear medicine examinations at a quaternary academic center.

**Materials and methods:**

In this IRB approved study, we retrospectively reviewed nuclear medicine examinations submitted for secondary interpretation by a pediatric radiologist with a nuclear medicine focus at our institution between 08/2019 and 08/2024. A single reviewer compared the primary and secondary reports to identify discrepancies that would likely impact clinical management, and discrepancies were confirmed by additional reviewers. Pediatric hematology/oncology faculty (*n*=29) and fellows (*n*=18) at our institution were surveyed to understand requests for, and the impact of, secondary interpretations. Results are summarized with descriptive statistics.

**Results:**

Three hundred fifty-eight examinations (median patient age=8 years) were included, 237 were ^18^F-FDG PET body scans, 97 were ^123^I-MIBG scans, and 24 were other examinations. Secondary interpretations yielded meaningful changes in 17% (60/358). Of these, 20% (12/60) changed from negative/normal to positive, 20% (12/60) changed from positive to negative/normal, and 57% (34/60) included additional positive findings/diagnoses. Sixteen survey responses (34%; *n*=16/47 response rate) were received, with providers indicating that secondary interpretations were clinically useful even when they agreed with the primary impression.

**Conclusion:**

Secondary interpretation of pediatric nuclear medicine examinations by pediatric radiologists with nuclear medicine focus resulted in changes that have potential impact on clinical management in 17% of cases. Secondary interpretations completely changed the impression regarding the presence or absence of malignant disease in 40% of these cases. Referring providers identified benefit in secondary interpretations even when they confirmed the primary impression.

**Supplementary Information:**

The online version contains supplementary material available at 10.1007/s00247-025-06441-w.

## Introduction

Radiologists at referral and academic medical centers are tasked with reviewing (“over-reading,” “providing secondary interpretation”) imaging examinations performed and previously interpreted at another facility for some patients who are transferred or seeking additional care. Literature from trauma centers suggests that approximately 50% of patients transferred to these centers have outside imaging that is over-read [1, 2]. More broadly, Rosenkrantz et al. reported that Medicare claims for secondary interpretations showed a compound annual growth of more than 20% from 2003 to 2016 and Lu et al. demonstrated that Medicare claims for secondary interpretations of CT examinations increased by 811% between 1999 and 2012 [3, 4].

Secondary interpretations of imaging examinations are requested for many reasons, such as disagreement with the primary interpretation, specific or additional questions that were not addressed in the primary interpretation, or increased provider confidence in interpretations by radiologists within the receiving health system [5, 6]. Performing secondary interpretations of imaging examinations results in a second professional charge to the payor or patient, which may not be reimbursed, increases the workload of radiologists at centers performing secondary interpretations, and may be duplicative rather than adding value to the health care system [5]. However, data suggest that secondary interpretations can be diagnostically contributory. For example, Karmazyn et al. reported significant differences in 16.4% of secondary interpretations of pediatric skeletal surveys [7].

Subspecialty or advanced certifications are available for multiple disciplines within radiology, but most imaging is interpreted by generalist radiologists or radiologists without subspecialization specific to the imaging examination [8]. This is true for pediatric and nuclear medicine imaging examinations. Subspecialty interpretation has been shown to be beneficial in identifying clinically relevant findings in a substantial fraction of previously interpreted pediatric imaging examinations. Eakins et al. found major discrepancies in 12.6% and 32.6% of secondarily interpreted pediatric neuroimaging and body imaging examinations respectively [9]. Additional studies have examined the impact of secondary interpretations in adult imaging subspecialties. To our knowledge, the impact of secondary interpretations in pediatric nuclear medicine has not been explored.

The purpose of our study was to understand the impact of secondary interpretations of pediatric nuclear medicine examinations at our institution. Specifically, we sought to evaluate how frequently secondary interpretations performed by subspecialty-certified pediatric radiologists with focused experience in pediatric nuclear medicine meaningfully differed from the outside interpretation. We also sought to collect data from local referring providers about why they order secondary interpretations.

## Materials and methods

### Study design

This was a HIPAA-compliant study approved by our institutional review board (IRB). The retrospective records review portion of this study was approved with a waiver of the requirement for documentation of informed consent. The prospective provider survey was approved with consent implied by survey completion.

### Case and data selection

Using an electronic medical record (Hyperspace; Epic Systems; Verona, WI) query, we identified nuclear medicine imaging examinations performed at outside institutions and submitted for secondary interpretation by the section of Pediatric Nuclear Medicine during the period August 19, 2019, and August 7, 2024. We included examinations for patients aged 17 years and younger, and examinations for which the primary report was available for review. The section of Pediatric Nuclear Medicine at our institution is staffed by board-certified pediatric radiologists with a special focus in pediatric nuclear medicine and who staff the section approximately 1 day (or more) per week. During the study period, a total of nine radiologists, two of whom had completed a 1-year Nuclear Radiology fellowship in addition to a pediatric radiology fellowship, interpreted imaging examinations in the section of Pediatric Nuclear Medicine.

### Case classification

A pediatric radiology fellow (P.H.) reviewed the primary and secondary interpretation reports for the identified nuclear medicine examinations, specifically focusing on the impression. During this review, the fellow determined whether the major findings/diagnoses in the impression were meaningfully different between the primary and secondary interpretations. At their discretion, the fellow reviewed the full report to better understand impression details. Report differences were considered “meaningful” if, based on an understanding of the clinical role of nuclear medicine in the disease for which the patient was imaged, the finding(s) were likely to alter clinical management or imaging follow-up. Meaningful differences were classified into one or more of eight categories (Table 1) and individual cases could be assigned to multiple categories. Categories were defined inductively and were developed directly from the data. Impression details that were unlikely to alter clinical management were disregarded.

**Table 1:** Categories of “Meaningful Change” between primary and secondary interpretations of nuclear medicine examinations

Category of change	Definition
1	Changed from negative/normal to positive diagnosis
2	Changed from positive diagnosis to negative/normal, or the positive diagnosis was downplayed
3	Changed from positive diagnosis to the same positive diagnosis with additional positive findings or upgraded diagnosis
4	Changed from multiple different positive findings to fewer (or a single) positive finding/diagnosis
5	Significant change in management/imaging follow-up recommendations detailed in the report
6	Meaningful increase in disease scoring/grading, potentially affecting clinical management
7	Meaningful decrease in disease scoring/grading, potentially affecting clinical management
8	Re-classified as a “nondiagnostic” study

For changes in disease scoring, meaningful change thresholds were defined based on implications for clinical care. ^18^F-FDG PET examinations where the primary and secondary reports both categorized the examination as Deauville 1, 2, or 3 (with absence of FDG-avid bone marrow lesions) were interpreted as “no change.” However, when one report classified the examination as Deauville 1, 2, or 3 and the other classified the examination as Deauville 4 or 5, this was considered a meaningful change as Deauville 4 and 5 lesions are managed differently in the treatment algorithm outlined in the most recent Oncology clinical practice guidelines from the National Comprehensive Cancer Network [10]. For Curie scoring of ^123^I-MIBG examinations, the thresholds for meaningful change were arbitrarily defined for this study with reference to the clinically meaningful threshold of Curie=2 [11]. When both the primary and secondary reports categorized the examination as Curie 1 or 2, this was considered “no change.” If both ^123^I-MIBG reports gave a Curie score >2, but the scores differed by less than 2 points, then this was considered “no change.” If both reports gave a Curie score >2, but the difference was more than half or more than doubled, then this was considered a meaningful change (for example, a Curie score of 5 changed to 10 would be meaningfully changed, while a Curie score of 12 changed to 18 would not be considered meaningfully changed).

### Case classification validation

Four pediatric radiologists who consistently read clinical nuclear medicine examinations at our institution and have 4, 13, 18, and 27 years of post-fellowship experience validated the report classification performed by the fellow. In this validation process, all pairs of primary and secondary reports classified by the fellow as meaningfully changed and an equal number of randomly selected, modality-matched, cases classified as not meaningfully changed were reviewed. Each pair of reports was reviewed by two radiologists who were blinded to the original classification by the fellow. Reviewing radiologists classified report pairs using the same system employed by the fellow (Table 1). Majority opinion (≥2 reviewers agreeing) among the fellow and two radiologists was used to assign the final classification (meaningfully changed or not meaningfully changed). Further, to validate the classification by the fellow from a clinical perspective, a board-certified pediatric oncologist reviewed all pairs of primary and secondary reports classified by the fellow as meaningfully changed and the classification assigned by the fellow. For expediency, this reviewer did not review a matched number of cases classified as not meaningfully changed.

### Additional data collection

Patient demographic data were extracted from the electronic medical record. For cases classified as meaningfully changed, the outside institution and primary interpreting radiologist were recorded when available. Additionally, the subspecialty of the primary interpreting radiologist was identified through a web search.

### Provider survey

To understand why providers request secondary interpretation of pediatric nuclear medicine examinations and how secondary interpretations impact clinical care, we surveyed 29 clinical pediatric oncology faculty and 18 pediatric hematology/oncology fellows at our institution. A 13-question survey was created in REDCap (Appendix 1) and distributed by email [12, 13]. The initial invitation to complete the survey was sent on April 22, 2025. A reminder was sent on May 14, 2025. The survey was closed on May 23, 2025. Eight items concerned why respondents order secondary interpretations, two items characterized provider risk tolerance [14], and we included three demographic assessment items. The risk tolerance items allow characterization of respondent risk tolerance as risk averse, risk seeking, or prospect theory concordant (meaning they only took a gamble to avoid a loss).

### Statistical methods

Fleiss’ kappa statistic was used to determine agreement between reviewers. Values >0.75 indicate excellent agreement beyond chance, values between 0.40–0.75 indicate fair to good agreement, and values <0.40 indicate poor agreement [15].

## Results

A total of 388 imaging examinations submitted for secondary interpretation were identified through the initial medical record search. Of these, 30 examinations were excluded due to missing primary reports, leaving 358 cases for inclusion. These 358 cases represented 290 individual patients, with a median age of 8 (range, 4–13) years. Most included examinations were ^18^F-FDG PET body scans (66%, 237/358) and ^123^I-MIBG scans (27%, 97/358) (Table 2).

**Table 2:** Types of nuclear medicine examinations for which secondary interpretations were performed and frequency of meaningful change in secondary interpretations. Results are presented as counts and percentages

Imaging examination	Count (% of 358)	Frequency of meaningful change (% of examination type)
^18^F-FDG PET body scan	237 (66.2%)	39 (16.5%)
^123^I-MIBG scan	97 (27.1%)	16 (16.5%)
^99m^Tc-MDP bone scan	10 (2.8%)	1 (10.0%)
^99m^Tc-MAG3 renal scan	6 (1.7%)	2 (33.3%)
^99m^Tc-mebrofenin HIDA scan	4 (1.1%)	1 (25.0%)
^68^Ga-DOTATATE PET scan	1 (0.3%)	0 (0.0%)
^18^F-FDG PET brain scan	1 (0.3%)	0 (0.0%)
Gastric emptying scan	1 (0.3%)	0 (0.0%)
^99m^Tc-pertechnetate Meckel scan	1 (0.3%)	1 (100.0%)

Initial review by the fellow identified meaningful changes between the primary and secondary interpretation in 22% (80/358) of cases. In the validation process, which included these 80 cases plus 80 matched cases with no meaningful change identified by the fellow, there was agreement on classification between all three radiologist reviewers for 100 (63% of 160) cases and agreement between ≥2 radiologist reviewers for 120 (75% of 160) cases (Kappa=0.48 [95% CI, 0.38–0.58.38.58], indicating fair to good agreement).

Of the 80 cases initially labeled as meaningfully changed by the fellow, both secondary radiologist reviewers agreed with the initial classification in 36 cases (45%), and one of the secondary radiologist reviewers agreed with the initial classification in 23 cases (29%), resulting in a final classification of meaningfully changed for 59 cases. The remaining 21 cases (26%) were re-classified as not meaningfully changed based on both secondary radiologist reviewers disagreeing with the initial classification by the fellow. The oncologist reviewer agreed with the fellow’s classification of meaningfully changed in a higher number of cases (66/80), reflecting a frequency of meaningful change among all cases of 18%. Given the lack of significant difference between this frequency and the frequency based on agreement among multiple radiologist reviewers, we report the radiologist-validated frequency going forward.

Among the 80 matched cases initially classified as not meaningfully changed by the fellow, both secondary reviewers agreed in 64 cases (80%), and one of the two secondary reviewers agreed in 15 cases (19%), confirming classification as not meaningfully changed for 79 cases. In only 1 case (1%), both secondary reviewers disagreed with the initial classification, resulting in this case being re-classified as meaningfully changed.

Based on the review and validation process, 17% (60/358) of secondary interpretations of pediatric nuclear medicine examinations performed at outside facilities were meaningfully changed from the primary interpretation. Among these 60 cases, the most common type of change was the inclusion of additional findings or diagnoses (57%, 34/60). The frequency of other types of changes, including change from positive to negative and from negative to positive, is detailed in Table 3.

**Table 3:** Frequencies of meaningful change categories. More than one category could be assigned to any individual case of meaningful change

Category of “Meaningful Change”	Total	/60
1. Changed from negative/normal to positive diagnosis	12	20%
2. Changed from positive diagnosis to negative/normal, or the positive diagnosis was downplayed	12	20%
3. Changed from positive diagnosis to the same positive diagnosis with additional positive findings or upgraded diagnosis	34	56.7%
4. Changed from multiple different positive findings to fewer (or a single) positive finding/diagnosis	11	18.3%
5. Significant change in management/imaging follow-up recommendations detailed in the report	7	11.7%
6. Meaningful increase in disease scoring/grading, potentially affecting clinical management	13	21.7%
7. Meaningful decrease in disease scoring/grading, potentially affecting clinical management	10	16.7%
8. Re-classified as a “nondiagnostic” study	3	5%

An example of interpretation change from “negative to positive” was:

*Primary interpretation* - “no evidence of FDG-avid recurrent or metastatic disease.”

**Fig. 1:**
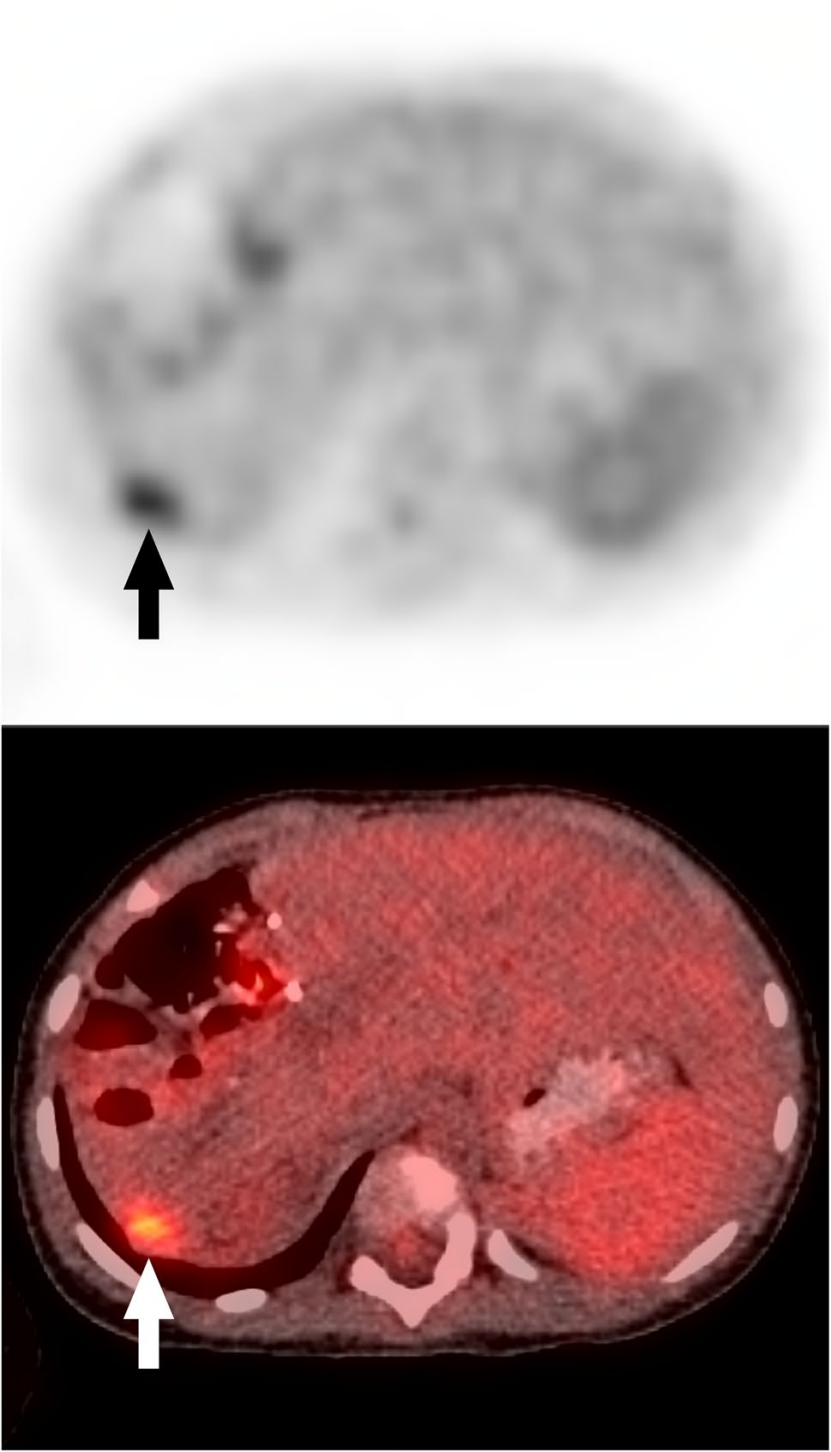
Axial PET and fused PET/CT images from ^18^F-FDG PET/CT in a 2-year-old girl with history of hepatoblastoma status post chemotherapy and partial hepatectomy with rising alpha fetoprotein. The primary interpretation reported “no evidence of FDG-avid recurrent or metastatic disease.” The secondary interpretation identified “focal abnormal FDG uptake in segment 7 of the liver compatible with recurrent/metastatic disease in context of rising AFP.” *Arrows* indicate the finding

*Secondary interpretation *(Fig. 1) - “Focal abnormal FDG uptake...in the liver compatible with recurrent/metastatic disease in context of rising AFP.” The secondary report also mentioned “abnormal uptake in the retroperitoneum and root of the mesentery.”

An example of change from “positive to negative” was:

*Primary interpretation* - “Area of FDG activity along the pylorus…given history of hepatoblastoma, metastatic implants not excluded…” The report recommended MRI/CT imaging.

**Fig. 2:**
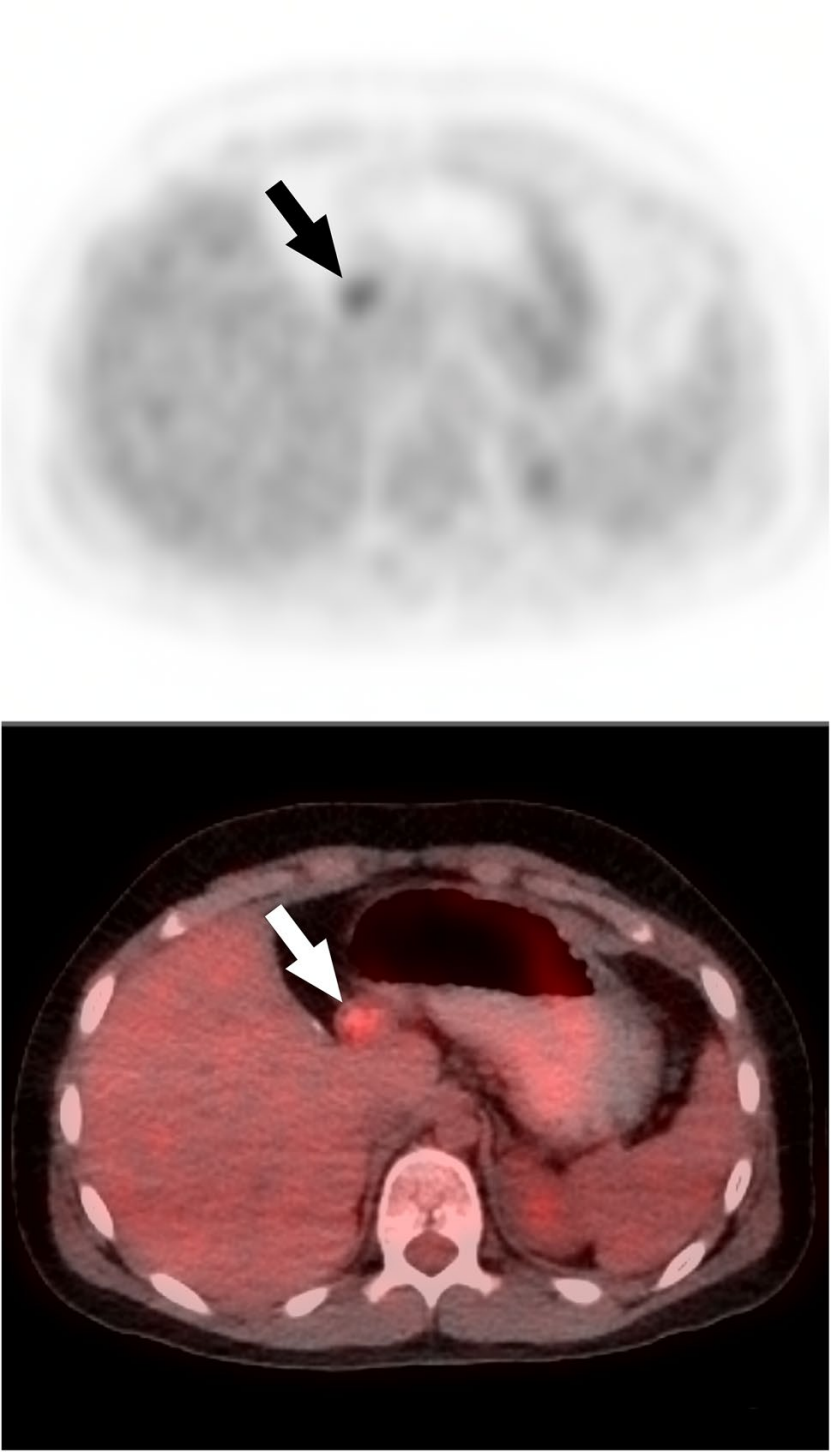
Axial PET and fused PET/CT images from ^18^F-FDG PET/CT in an 11-year-old boy with hepatoblastoma. The primary interpretation reported an “area of FDG activity along the pylorus… metastatic implants not excluded…” and recommended further diagnostic MRI or CT. The secondary interpretation reported “no findings of recurrent or metastatic disease” and “upper abdominal activity described on the outside report reflects activity associated with the pylorus and is not concerning for disease.” *Arrows* indicate the finding

*Secondary interpretation *(Fig. 2) - “No findings of recurrent or metastatic disease” and “upper abdominal activity reflects physiologic activity.”

### Frequency of meaningful change by modality

Among modalities, the frequency of meaningful change was 17% for both ^18^F-FDG PET body scans (39/237) and ^123^I-MIBG scans (16/97). Meaningful changes were less common among other modalities (Table 1).

### Specialty of physicians performing primary interpretations

The 60 cases that had meaningfully changed secondary interpretations were primarily interpreted by 52 unique physicians, 56% (28/50) of whom were subspecialized in nuclear medicine, 22% (11/50) of whom were subspecialized in pediatric radiology, 12% (6/50) of whom were general radiologists, 8% (4/50) of whom were subspecialized in body radiology, and 2% (1/50) of whom were subspecialized in interventional radiology.

### Referring provider survey results

Sixteen survey responses were received, reflecting a 34% (16/47) response rate. Respondents included current fellows (50%, 8/16), faculty with less than 1 year of experience (25%, 4/16), faculty with less than 3 years of experience (13%, 2/16), and faculty with greater than 15 years of experience (13%, 2/16). Among respondents, 31% (5/16) were general oncologists with no specific focus, 25% (4/16) had solid tumor focus, 19% (3/16) had neuro-oncology focus, 13% (2/16) had leukemia focus, and 13% (2/16) had lymphoma focus. Only 19% (3/16) of respondents had previously had a faculty appointment outside of our institution at some point post-fellowship.

Based on our survey items concerning respondent risk tolerance, when presented with the two scenarios that measured willingness to gamble on behalf of patients, most providers (69%; 11/16) were prospect theory concordant, meaning they only took a gamble to avoid a loss. This included 5 fellows and 6 faculty. Three providers (19%, 1 fellow and 2 faculty) were risk averse, and 2 providers (13%, both fellows) were risk seeking.

For new patient consults, most respondents (88%, 14/16) stated that they “almost always” request secondary interpretations of nuclear medicine examinations; the remaining 2/16 respondents said they request secondary interpretations 50% and 25% of the time respectively. For existing patients (including those in follow-up), 69% (11/16) of respondents stated that they “almost always” request secondary interpretations, 13% (2/16) said they request secondary interpretations “75% of the time,” 13% (2/16) said “50% of the time,” and 6% (1/16) said “25% of the time.”

To “Confirm an outside read that directly impacts therapy” was the most common reason for requesting a secondary interpretation for 56% (9/16) of respondents. “Changes management” (13%, 2/16), “Protocol requirement” (6%, 1/16), “Changes my confidence in management” (6%, 1/16), “Not confident in outside read” (6%, 1/16), and “To avoid repeating the exam because of risk (e.g. radiation, sedation, etc.) to the patient” (6%, 1/16) were the most common reasons for requesting secondary interpretations for the remaining respondents. The reasons for requesting secondary interpretations that were most frequently ranked among the top three by respondents were “Confirm an outside read that directly impacts therapy” (*n*=10), “To avoid repeating the exam because of risk to the patient” (*n*=7), “To have formal documentation in the local chart” (*n*=5), and “Not confident in outside read” (*n*=5).

Less than half of respondents (44%, 7/16) indicated that the specific imaging modality influences their likelihood of requesting a secondary interpretation. Free-text comments in support of these responses suggest that respondents were more likely to request secondary interpretations for PET/CT or MRI, and less likely to request secondary interpretations for CT.

When asked to estimate the frequency with which a secondary interpretation of an outside examination differs significantly from the primary interpretation, 38% (6/16) responded “5–25%,” 25% (4/16) responded “26–50%,” 25% (4/16) responded “51–75%,” and 13% (2/16) responded “I don’t know.” When asked to estimate the frequency with which a secondary interpretation has a significant effect on clinical management, there was similar variability: 6% (1/16) responded “<5%,” 25% (4/16) responded “5–25%,” 13% (2/16) responded “26–50%,” 25% (4/16) responded “51–75%,” 6% (1/16) responded “76–100%,” and 25% (4/16) responded “I don’t know.” All respondents indicated that secondary interpretation is still useful even if the secondary interpretation agrees with the primary interpretation. Free-text comments in support of these responses generally reflected that secondary interpretations provide confidence in management because they are confirmed by radiologists that the provider trusts (Table 4).

**Table 4:** Thematic summary of free text survey responses

Comment theme	Count
Greater confidence in local interpretation	8
Increased confidence in making treatment/management decision(s)	7
Provides an assessment of the quality of interpretation(s) coming from the outside institution (can they be trusted going forward?)	2
Opportunity to discuss interpretation with local radiologist	1

## Discussion

Secondary interpretations of pediatric nuclear medicine examinations led to meaningful changes in the report impression and/or follow-up recommendations in 1 in 6 cases when reviewed by a subspecialty service at our quaternary academic medical center. The most common changes were the inclusion of additional findings or diagnoses (57%). However, 40% of changes, accounting for 7% of all cases, reflected a complete shift in interpretation from either positive to negative or negative to positive.

While the literature is lacking related to the frequency and impact of secondary interpretations specific to pediatric nuclear medicine, studies have examined the impact of secondary interpretations in other areas of pediatric imaging. Eakins et al. found major discrepancies in 13% and 33% of secondarily interpreted pediatric neuroimaging and body imaging examinations respectively [9]; Karmazyn et al. reported significant differences in 16% of secondary interpretations of pediatric skeletal surveys [7] and in a meta-analysis of 29 studies, Rosenkrantz et al. reported a discrepancy frequency of 35.5% for pediatric imaging [16]. In a mixed sample of pediatric and adult imaging, Hatzoglou et al. evaluated interpretations by oncological neuroradiologists and felt that care was impacted by secondary interpretation in 15% of the cases [17]. The frequency of meaningful change reported in these studies is similar to that in our study.

Other studies have looked at the impact of secondary interpretations of nuclear medicine imaging examinations in adults. Ulaner et al. found that 13% (31/240) of secondary ^18^F-FDG PET body scan reports had at least one discordant opinion of malignancy [18]. This frequency is similar to the frequency of meaningful change in ^18^F-FDG PET body scans in our sample (17%).

Providing secondary interpretations of imaging examinations results in a second professional charge to the payor or patient. At our institution, examinations performed at outside institutions and submitted for secondary interpretation are loaded into the PACS system under order/procedure types specific to the examination type with a tag that bills only the professional fee when the interpretation of the examination is finalized. Examination-specific orders carry the relevant CPT codes for billing, requiring no post hoc coding action. For patient accounts that are self-pay, government, or Medicaid, coders apply two modifiers: “26” indicating that the professional fee only is billed and “77” indicating that the charge is for a second interpretation of an examination that was already interpreted. While we did not review reimbursement for the pediatric nuclear medicine examinations included in this study, a prior study by Seghers et al. showed that at their quaternary pediatric hospital, 17% of all secondary interpretations, regardless of modality, were not charged, and 40.6% of charged secondary interpretations were reimbursed [6]. This compared to a reimbursement frequency of 42.8% for primary interpretations over the same period.

In addition to quantifying the frequency of meaningful change in secondary interpretations, we sought to characterize the rationale for, and perceived value of, secondary interpretations among our referring oncology practitioners. Their responses suggest that they mainly request secondary interpretations to confirm primary interpretations that directly impact therapy, and to avoid repeating the examination. Responses also suggest that much of the value of secondary interpretations comes from increasing confidence in the interpretation and their clinical management. While respondents had variable estimates of the frequency with which secondary interpretations significantly varied from the outside read, as well as how often secondary interpretations have a significant effect on clinical management, all respondents indicated there was value in the secondary interpretation even if it fully agreed with the primary interpretation. This reflected the fact that respondents felt that confirmatory interpretations provide confidence in management because they come from radiologists that the provider trusts, a finding that builds on the prior study by Rizzo et al. which demonstrated that oncologists prefer oncologic imaging reports that are prepared by subspecialized radiologists versus general radiologists [19]. This finding also aligns with our finding that most survey respondents were either risk averse (never took a gamble) or only took a gamble to avoid a loss. This suggests that while 83% of secondary interpretations in our study did not result in a meaningful change to the report, these were not necessarily without purpose as they may have provided confidence in clinical management.

Our study is limited by the fact that it reflects the experience of a single quaternary academic institution where pediatric nuclear medicine examinations are read by a subspecialized group. Furthermore, our study only examined the frequency of changes in reports that were likely to alter clinical management or imaging follow-up and did not seek to define which interpretation was “correct.” While this prevents us from assessing the accuracy of secondary versus primary interpretations, our purpose was to understand whether there was potential value in the secondary read process – which there appears to be. Additionally, our sample was largely comprised of secondary interpretations of ^18^F-PET body scans and ^123^I-MIBG scans, which comprised over 90% of our cases. This sample bias likely reflects the type of patients transferred to our institution and the type of nuclear medicine examinations performed on these patients. As a result, we cannot make firm conclusions about other pediatric nuclear medicine imaging examinations. Our study is also limited by the fact that only a subset of reports, including all reports with an identified significant change, were reviewed by physicians experienced in pediatric nuclear medicine to verify the presence or absence of meaningful changes assigned by the fellow. However, while 21/80 cases initially classified as meaningfully changed by the fellow were re-classified to not meaningfully changed during validation, only one of the matched cases initially classified as not meaningfully changed by the fellow was re-classified to meaningfully changed. This suggests that the fellow was sensitive to, and rarely missed, a meaningful change. We suspect this augmented sensitivity may reflect the fact that the fellow was seeking significant change in the context of this study. Additionally, the survey component of our study is limited by the fact that it only reflects the opinion of providers at our institution, the response rate was low, and our sample was heavily biased toward fellows. Finally, the rationale for the secondary interpretation request is not typically included in the history or order details at our institution and thus this must be inferred from the survey responses.

## Conclusion

Secondary interpretations of pediatric nuclear medicine examinations by pediatric radiologists with focused experience in pediatric nuclear medicine resulted in a change that likely affected clinical management 17% of the time. A substantial fraction of these changes were complete changes in interpretation from benign/negative to the presence of malignant disease, or vice versa. Referring providers find value in secondary interpretations even when they agree with the primary interpretation, as this provides confidence in both the interpretation and resulting clinical management. Our findings highlight the clinical importance of secondary interpretations of pediatric nuclear medicine examinations, particularly when performed by subspecialty-trained pediatric radiologists.

## Supplementary Information

Below is the link to the electronic supplementary material.

Supplementary file1 (PDF 49.8 KB)

## Data Availability

The data that support the findings of this study are not openly available due to reasons of sensitivity and are available from the corresponding author upon reasonable request with required redactions to protect patient confidentiality.
