# Supplementary material for: Discrepancies between primary and secondary interpretations of pediatric nuclear medicine imaging examinations
Source: Pediatr Radiol. 2025 Dec 4;56(2):464–72. doi: 10.1007/s00247-025-06441-w (PMC12881077; doi:10.1007/s00247-025-06441-w)
Supplement: Supplementary file 1 — Supplementary file1 (PDF 49.8 KB) [file 247_2025_6441_MOESM1_ESM.pdf]

Clinical Value of Overreads - Provider Survey Questions

Please complete the survey below.

The purpose of this survey is to understand why CBDI providers order overreads (CCHMC reads of outside exams). Your answers are anonymous and confidential. Thank you for sharing your opinion with us.

Thank you!

For new patient consults, how often do you request overreads of nuclear medicine studies performed at outside hospitals?

- ◯ Almost never
- ◯ ~25% of the time
- ◯ ~50% of the time
- ◯ ~75% of the time
- ◯ Almost always

For existing patients (including those in follow-up), how often do you request overreads of nuclear medicine studies performed at outside hospitals?

- ◯ Almost never
- ◯ ~25% of the time
- ◯ ~50% of the time
- ◯ ~75% of the time
- ◯ Almost always

From the following reasons, select the 3 MOST COMMON reasons for which you request an overread of a nuclear medicine examination performed at an outside hospital?

	1st most common	2nd most common	3rd most common
Protocol requirement	◯	◯	◯
To have formal documentation in the local chart	◯	◯	◯
Changes management	◯	◯	◯
Changes my confidence in management	◯	◯	◯
Confirm an outside read that directly impacts therapy	◯	◯	◯
Parent/guardian request	◯	◯	◯
Not confident in outside read	◯	◯	◯
Reputation of the outside institution	◯	◯	◯
Disease type	◯	◯	◯
Disease severity/stage	◯	◯	◯
To avoid repeating the exam because of risk (e.g. radiation, sedation, etc.) to the patient	◯	◯	◯
To avoid repeating the exam because of cost to the patient	◯	◯	◯
Missing information relevant to management	◯	◯	◯
Unclear or ambiguous outside interpretation	◯	◯	◯

Other	◯	◯	◯

Please list the other reason you request overreads of nuclear medicine examinations preformed at outside hospitals

____

If the outside study was interpreted at a children's hospital or by a pediatric radiologist how likely are you to request an overread at CCHMC?

- ◯ Almost never
- ◯ ~25% of the time
- ◯ ~50% of the time
- ◯ ~75% of the time
- ◯ Almost always

Does the imaging modality (e.g. CT vs. MRI vs. PET vs. other) influence your likelihood of requesting an overread at CCHMC?

- ◯ Yes
- ◯ No

How?

____

In your experience, approximately what percentage of the time does an over-read of an outside study by a CCHMC radiologists differ significantly from the outside interpretation?

- ◯ < 5%
- ◯ 5-25%
- ◯ 26-50%
- ◯ 51-75%
- ◯ 76-100%
- ◯ I don't know

In your experience, approximately what percentage of the time does an over-read of an outside nuclear medicine study by a CCHMC radiologist have a significant effect on how you manage the patient?

- ◯ < 5%
- ◯ 5-25%
- ◯ 26-50%
- ◯ 51-75%
- ◯ 76-100%
- ◯ I don't know

If the CCHMC over-read says exactly the same thing that the outside read said, is this useful to you?

- ◯ Yes
- ◯ No

Why?

____

DEMOGRAPHICS

For how many years have you been practicing after completing your pediatric oncology fellowship?

- ◯ Current fellow
- ◯ < 3
- ◯ 3-5
- ◯ 6-10
- ◯ 10-15
- ◯ >15

What is your primary focus of practice?

- ◯ Neurooncology
- ◯ Solid tumor
- ◯ Leukemia
- ◯ Lymphoma
- ◯ General/No focus

Since completing your pediatric oncology fellowship have you had a faculty appointment outside of CCHMC?

- ◯ Yes
- ◯ No

For the following 2 questions, consider a patient with cancer who is either relatively "healthy" or relatively "sick"

How would you handle the following decision? Choose between two new therapies for a relatively healthy patient:

- ◯ A 100% chance of living 5 years MORE than the average patient AND a 0% chance of living 0 years MORE than the average patient.
- ◯ A 50% chance of living 10 years MORE than the average patient AND a 50% chance of living 0 years MORE than the average patient.

How would you handle the following decision? Choose between two new therapies for a relatively sick patient:

- ◯ A 100% chance of living 5 years LESS than the average patient AND a 0% chance of living 10 years LESS than the average patient.
- ◯ A 50% chance of living as long as the average patient AND a 50% chance of living 10 years LESS than the average patient.
